# Tuning Optical and Granulometric Properties of Gold Nanostructures Synthesized with the Aid of Different Types of Honeys for Microwave-Induced Hyperthermia

**DOI:** 10.3390/ma12060898

**Published:** 2019-03-18

**Authors:** Anna Dzimitrowicz, Piotr Cyganowski, Piotr Jamroz, Dorota Jermakowicz-Bartkowiak, Malgorzata Rzegocka, Agnieszka Cwiklinska, Pawel Pohl

**Affiliations:** 1 Department of Analytical Chemistry and Chemical Metallurgy, Faculty of Chemistry, Wroclaw University of Science and Technology, Wybrzeze St. Wyspianskiego 27, 50-370 Wroclaw, Poland; piotr.jamroz@pwr.edu.pl (P.J.); rzegocka.malgorzata@gmail.com (M.R.); a.cwiklinska328@gmail.com (A.C.); pawel.pohl@pwr.edu.pl (P.P.); 2 Department of Polymer and Carbonaceous Materials, Faculty of Chemistry, Wroclaw University of Science and Technology, Wybrzeze St. Wyspianskiego 27, 50-370 Wroclaw, Poland; piotr.cyganowski@pwr.edu.pl (P.C.); dorota.jermakowicz-bartkowiak@pwr.edu.pl (D.J.-B.)

**Keywords:** food products, green synthesis, nanostructures, phenolics, reducing sugars

## Abstract

Size-controlled gold nanoparticles (AuNPs) were synthesised with solutions of three types of Polish honeys (lime, multiflower, honeydew) and used in microwave-induced hyperthermia cancer treatment. Optical and structural properties of nanostructures were optimized in reference to measurements made by using UV/Vis absorption spectrophotometry (UV/Vis), transmission electron microscopy (TEM) supported by energy-dispersive X-ray spectroscopy (EDX), X-ray diffraction (XRD), and attenuated total reflectance Fourier transformation infrared spectroscopy (ATR FT-IR). In addition, concentrations of reducing sugars and polyphenols of honeys applied were determined to reveal the role of these chemical compounds in green synthesis of AuNPs. It was found that the smallest AuNPs (20.6 ± 23.3 nm) were produced using a 20% (*w*/*v*) multiflower aqueous honey solution and 25 mg·L^−1^ of Au(III) ions. These AuNPs were then employed in microwave-induced hyperthermia in a system simulating metastatic tissues. This research illustrated that AuNPs, as produced with the aid of a multiflower honey solution, could be suitably used for microwave-induced heating of cancer. A fluid containing resultant Au nanostructures, as compared to water, revealed facilitated heating and the ability to maintain a temperature of 45 °C required for hyperthermia treatment.

## 1. Introduction

Gold nanoparticles (AuNPs) are classified as three-dimensional nanomaterials (NMs) in which at least one of the dimensions in the structure is less than 100 nm [1]. Due to their high surface to volume ratio, AuNPs display unique conductive, catalytic, optical, structural, thermal, and thermoplasmonic properties [2,3,4,5,6,7]. For that reason, they are widely utilized as conductors for flexible electronics [8], catalysts for polymerization of alkylosilanes [9], detectors for severe acute respiratory syndrome (SARS) coronavirus [10], contrast agents in X-ray imaging [11], vehicles for drug delivery [12], agents in photo-induced hyperthermia [13], and in microwave-induced hyperthermia treatment of cancer [14].

Microwave-induced hyperthermia treatment of cancer has gained a lot of interest in the past several years. This treatment is recognized as efficient and non-invasive [15], being successfully applied for the treatment of metastatic tissues in bones [16], breast [17,18,19], prostate [20], bladder [14], brain [15], and liver [21]. Since hyperthermia-based medical procedures rely on the susceptibility of metastatic tissues to be destroyed at elevated temperatures (41–47 °C) [22], the increase in the thermal response of the area surrounding a tumour may lead to more effective treatment. In microwave-induced hyperthermia procedures, this is achieved by applying nanofluids (NFs) that usually contain metallic nanostructures with ability to be electromagnetically excited. The most common NFs are Fe_3_O_4_-based and are effective in the hyperthermia treatment of bone metastases [16]. AuNPs are usually applied in sophisticated procedures of photo-induced hyperthermia due to the plasmonic effect that they cause [13]. However, based on our previous studies [23,24], AuNPs and PtNPs have the extraordinary ability to transfer and accumulate heat, even at ultra-trace concentrations. Therefore, it can be hypothesized that the application of functionalized AuNPs in microwave-induced hyperthermia treatment of cancer may also be very effective.

The wide-range of biomedical applications of AuNPs are related not only to their optical and structural properties, but could also be influenced by toxicity and the chemical composition of reducing and capping agents involved in their synthesis. Although many methods were described for the production of AuNPs, green methods are a promising alternative to well-known chemical methods [25]. In green synthesis, naturally occurring substances are used as reducing and stabilizing agents during AuNPs production instead of strong, and usually toxic, chemicals. For that reason, synthesized AuNPs are biogenic and biocompatible [26,27,28]. There are a variety of natural products reported to be suitable for green synthesis of AuNPs. Accordingly, aqueous leaf extracts prepared from *Mentha piperita* [28], *Melissa officinalis* [28], *Salvia officinalis* [28], *Terminalia catappa* [29], *Rosa rugosa* [30], *Hibiscus rosa sinensis* [31], *Cinnamomum camphora* [32], *Eucalyptus globulus* [33], *Rosmarinus officinalis* [33], *Ziziphus zizyphus* [34], and *Plumbago zeylanica* [35], aqueous extracts originated from blackberries, blueberries, and pomegranates [36], in addition to essential oils collected from *Eucalyptus globulus* [33], *Rosmarinus officinalis* [33], *Anacardium occidentale* [37], *Curcuma pseudomontana* [38], and some food products, including *Punica granatum* juices [39], coffee Arabica infusions [40], and cacao powder beverages [41] were involved in the production of AuNPs. To the best of our knowledge, only a few research groups have attempted to produce honey-based NFs containing Au nanostructures [42,43,44].

Honey is obtained by several from about 20,000 species of bees from nectar, pollen, and honeydew [45]. A large variety of plant species from which bees might collect nectar, pollen, or honeydew, as well as apiary location, and environmental conditions means that several types of honeys can be distinguished [46]. Among them, the most popular are flower, e.g., multi-flower, rapeseed, buckwheat, lime, or heather, and honeydew honeys made from coniferous, leaf, and fir honeydew [47]. The botanical provenience of the nectar, pollen, and honeydew, as well as environmental pollution, influence the chemical composition of honey [48], which can contain more than 400 different compounds [48]. In addition to water, which content in honey is less than 20% (*w*/*v*) [45], there are many others substances, namely sugars (e.g., glucose, D-fructose, maltose) [49], aliphatic acids (e.g., formic acid, tartaric acid, citric acid, benzoic acid) [50], phenolics (e.g., quercetin, kaempferol, luteoil, rutin) [51], terpenes (e.g., monoterpenes) [52], ketones (e.g., acetoin) [53], vitamins (e.g., ascorbic acid, niacin, pyridoxine) [45], and proteins (e.g., alanine, arginine, serine, proline, phenylalanine) [45].

Application of honey in green synthesis of AuNPs is particularly interesting due to a large variety of honey types that might be used for that purpose, and their differentiated chemical compositions. For example, Philip [42] utilized an Indian natural honey solution for the production of spherical (~15 nm in size) AuNPs and suggested that fructose worked as a reducing agent for this. Snitka et al. [43] reported green synthesis of irregular-shaped ~30 nm Au nanostructures using a Lithuanian natural honey solution. Finally, Sreelakshimi et al. [44] also presented the application of an Indian honey for formation of 10 nm, spherical AuNPs with antibacterial activity towards different microorganisms such as pathogenic bacteria and fungus from Candida species.

In this work, the possibility of applying three different types of Polish honeys, i.e., lime, multi-flower, and honeydew, for green synthesis of size-defined AuNPs with a possible use in microwave-induced hyperthermia treatment of cancer was examined. By tuning the type of applied aqueous honey solutions, the honey concentration in these solutions, as well as the concentration of Au(III) ions added to these aqueous solutions, optimal conditions, at which the smallest in size AuNPs were produced, were found by applying UV/Vis absorption spectrophotometry (UV/Vis). Then, nanofluids (NFs) containing AuNPs synthesized under these optimal conditions were characterized using transmission electron microscopy (TEM) supported by energy-dispersive X-ray spectroscopy (EDX), and X-ray diffraction (XRD). Next, attenuated total reflectance Fourier transform-infrared spectroscopy (ATR FT-IR) was applied to identify chemical compounds of honey solutions responsible for the production of AuNPs. Particularly, to reveal the role of reducing sugars and phenolic compounds in examined green synthesis of Au nanostructures, total concentrations of reducing sugars and phenolic compounds in applied honey solutions were determined using the Bertrand’s method and the Folin-Ciocalteu assay, respectively. Furthermore, AuNPs of defined optical and granulometric properties were tested in a microwave radiation field, simulating procedures of hyperthermia treatment of cancer.

## 2. Materials and Methods

### 2.1. Reagents and Solutions

Three types of Polish honeys, i.e., lime, multi-flower, and honeydew, were purchased in a local supermarket. Chloroauric acid tetrahydrate (HAuCl_4_·4H_2_O) was from Avantor Performance Materials (Gliwice, Poland). The Bertrand’s solutions, i.e., I (CuSO_4_), II (a mixture of NaOH and C_4_H_4_KNaO_6_), and III (a mixture of Fe_2_(SO_4_)_3_ and H_2_SO_4_), were prepared using proper reagents taken from Sigma-Aldrich (Steinheim, Germany). The Folin–Ciocalteu reagent (3H_2_O·P_2_O_5_·14WO_3_·4MoO_3_·10H_2_O) and KMnO_4_ were obtained from Sigma-Aldrich as well. Re-distilled water was used throughput. All reagents were of analytical grade or better.

### 2.2. Honey-mediated Green Synthesis of AuNPs

**Figure 1:**
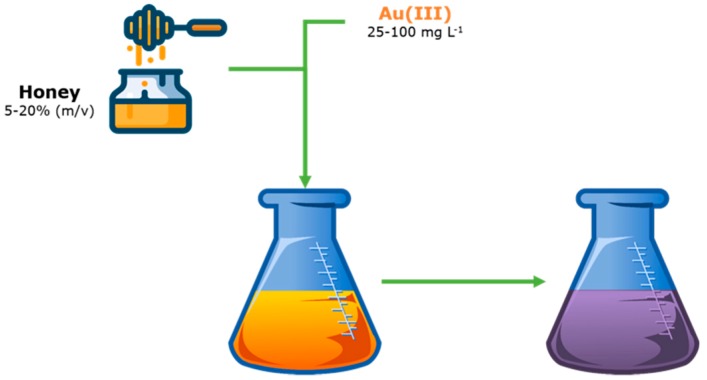
Green synthesis of gold nanoparticles (AuNPs).

As the AuNPs precursor, a 500 mg·L^−1^ stock solution of Au(III) ions was prepared by dissolving an appropriate amount of solid HAuCl_4_·4H_2_O in 250 mL of re-distilled water. Next, as shown in Figure 1, a certain volume of the resultant stock solution of Au(III) ions was mixed with a certain volume of aqueous honey solutions at concentrations of 5, 10, 15, or 20% (*w*/*v*); the final concentration of Au(III) ions in the resulting mixed solution was 25, 50, and 100 mg·L^−1^ (Figure 1). AuNPs synthesis took place at room temperature until the colour of mixed turned to ruby-red. Afterwards, obtained NFs were stored in the fridge at 4 °C for subsequently analyses.

### 2.3. Characterization of AuNPs Produced under Optimal Conditions

To find optimal conditions for the synthesis of the smallest in size AuNPs, the effect of the type of honey (lime, multiflower, honeydew), its concentration in prepared solutions (5, 10, 15, and 20% (*w*/*v*)), and the concentration of Au(III) ions (25, 50, and 100 mg·L^−1^) in mixed solutions on wavelength position at maximum (λ_max_) of the localized surface plasmon resonance (LSPR) absorption band of AuNPs was assessed. Optical properties of synthesized AuNPs were assessed using UV/Vis absorption spectrophotometry with a Specord 210 Plus spectrophotometer (Analytik Jena AG, Jena, Germany). These UV/Vis absorption spectra were recorded 72 h after AuNPs synthesis in the spectral range of 300–1100 nm, with a step of 0.1 nm, and a scanning speed of 20 nm·s^−1^. Re-distilled water was applied to zero the spectrophotometer. UV/Vis absorption spectra of raw honey solutions, and Au(III) ions solutions were also determined.

Granulometric properties of AuNPs produced under optimal synthesis conditions were assessed using various techniques. For the determination of size, shape, and elemental composition of AuNPs, TEM (Tecnai G^2^20 X-TWIN, FEI, Hillsboro, OR, USA) equipped with an EDX instrument (FEI, Hillsboro, OR, USA) was applied. To carry out these analyses, resultant NFs were diluted 10-fold, placed onto a Cu grid (CF 400 Cu-UL, Electron Microscopy Sciences, Hatfield, PA, USA), and evaporated under ambient air. After that, the Cu grid was placed in a TEM chamber. Size and shape distributions of AuNPs were assessed on the basis of 65 single NPs.

XRD was used to estimate the crystalline structure of AuNPs synthesized under optimal conditions. Measurements were performed in the symmetric Θ/2Θ Bragg-Brentano geometry, using a X-Pert Pro MPD diffractometer (Malvern Panalytical, Malvern, UK) equipped with a CuK α radiation source (λ = 0.15406 nm). They were carried out after placing resultant NFs onto a poly(methylmetacrylate) holder and left to dry in ambient air.

### 2.4. Qualitative Analyses of Chemical Compounds in Honey Solutions

ATR FT-IR spectroscopy was applied for the identification of chemical compounds present in aqueous honeys solutions used for synthesis of AuNPs. ATR FT-IR spectra were acquired in the range from 4000 to 370 cm^−1^. 20% (*w*/*v*) aqueous honeys solutions and resultant NFs were analyzed. A Vertex 70v ATR FT-IR spectrophotometer (Bruker, Bremen, Germany) equipped with a diamond ATR cell was applied for that purpose.

### 2.5. Quantitative Analyses of the Reducing Sugars and Phenolic Compounds Contained in Aqueous Honey Solutions

The Bertrand method was applied to determine amounts of reducing sugars in honey solutions following description given in references [54,55]. Briefly, 2 mL of a given 1% (*w*/*v*) honey solution was mixed with 20 mL of the Bertrand’s I solution and 20 mL of the Bertrand’s II solution. Then, the resultant mixture was boiled for 4 min, and then cooled down under cold tap water. The Cu_2_O precipitation formed was washed several times with warm re-distilled water by decanting over a Pyrex^®^ Gooch crucible (Sigma-Aldrich, Steinheim, Germany). In the next step, washed Cu_2_O was treated with 20 mL of the Bertrand’s III solution. The resultant solution was immediately titrated by a titrating KMnO_4_ solution. Titration was carried out until the titrated solution turned to bright pink. One mL of the titrating KMnO_4_ solution is associated with 6.537 g of reduced Cu(II) ions [55]. The amount of reducing sugars in honeys solution was calculated as follows: reducing sugars (in mg) = 0.564 × reduced Cu(II) ions (mg).

The Folin-Ciocalteu assay was used to assess the concentration of phenolic compounds before and after the addition of Au(III) ions into the given honey solutions [55]. Briefly, 2.5 mL of a 10-fold diluted solution of the Folin-Ciocalteu reagent was mixed with a 0.5 mL of a given 20% (*w*/*v*) honey solution. The so-prepared solution was incubated for 15 min at 50 °C. After 15 min, it was cooled down in an ice bath for 4 min. Next, absorbance related to the phosphotungstic-phosphotomolybdenium complex formed was measured at 765 nm using a Specord 210 Plus spectrophotometer (Analytik Jena AG, Jena, Germany). To zero the instrument, re-distilled water was applied. Results associated with the concentration of phenolic compounds in analyzed aqueous honey solutions were expressed as gallic acid equivalents (GAE) and given in mg of GAE per L. The calibration curve for GA was acquired within the concentration range of 10 to 150 mg·L^−1^, treating all standard solutions following the above-described procedure.

### 2.6. Thermal Behaviour of AuNPs in a Microwave Radiation Field

AuNPs synthesized with the aid of the selected honey solution were purified using dialysis tubing cellulose membranes (molecular weight cut-off 14 kDa, Sigma Aldrich, Steinheim, Germany). There was 10 mL of the so-obtained NF transferred into a 20 mL volumetric flask and 2-fold diluted with re-distilled water. Afterwards, this solution was introduced into a glass tubular reactor and placed in an ERTEC, model 02-02 microwave reactor (Ertec, Wroclaw, Poland). The procedure of heating was experimentally developed so as to heat the AuNPs-containing NF to 45 °C within 6 min. Then, temperature was maintained for another 4 min, applying a power of 15 W. The experiment was also carried out under the same conditions, but using water as a control. Temperature of each liquid was monitored every second and the heating rate was calculated using the simplified Newton’s law of heating, i.e., dT(*t*)/d*t* = k(h)∙∆T(*t*), where T(*t*) is temperature at a given time; k(h) is the heating rate (s^−1^), and ∆T(*t*) is a difference in temperature over time *t* [56].

## 3. Results and Discussion

### 3.1. Visual Observations of AuNPs Formation Followed by Determination of AuNPs Optical and Granulometric Properties

As compared to the yellowish colour of AuNPs precursor solutions, colloidal suspensions of AuNPs exhibit a ruby-red colour [55]. For that reason, the first indication that AuNPs were fruitfully synthesized with the aid of honey solutions was the change of colour of the mixed solutions from yellowish (before addition of a solution of Au(III) ions to a given honey solution) to ruby-red (after addition of the solution of Au(III) ions). Appearance of such a ruby-red colour in the mixed solution containing Au(III) ions at 100 mg·L^−1^ and 20% (*w*/*v*) of honeydew honey was observed after 5 min. On the other hand, for a lime tree honey and multi-flower honey, the mentioned ruby-red colour appeared after 30 and 35 min, respectively, for the mixed solution consisting 100 mg·L^−1^ of Au(III) ions and 20% (*w*/*v*) of honeys. In addition, it was noted that the formation of AuNPs, emanated by the appearance of the ruby-red colour of mixed solutions, strongly depended on the type of honey used for synthesis, as well as the concentration of honey, and the concentration of Au(III) ions in resultant mixed solutions. Accordingly, differences observed in time after which the ruby-red colour appeared in mixed solutions were directly related to differences in the concentration of chemical compounds, namely reducing sugars and polyphenols, that were responsible for the reduction of Au(III) ions and the stabilization of AuNPs formed in these conditions [43].

UV/Vis absorption spectrophotometry was applied to corroborate all visual observations, and to assess optical and granulometric properties of resultant AuNPs. Localized surface resonance of plasmonic AuNPs was responsible for their optical properties in visible light [57]. For colloidal suspension of AuNPs, the absorption band of their localized surface plasmon resonance (LSPR) is situated in the range from 520 to 550 nm [58]. Furthermore, as the size of AuNPs increases, the position of the LSPR absorption band shifts towards longer wavelengths, absorbing red light and reflecting blue light, as described by the Mie’s scattering theory [59].

**Figure 2:**
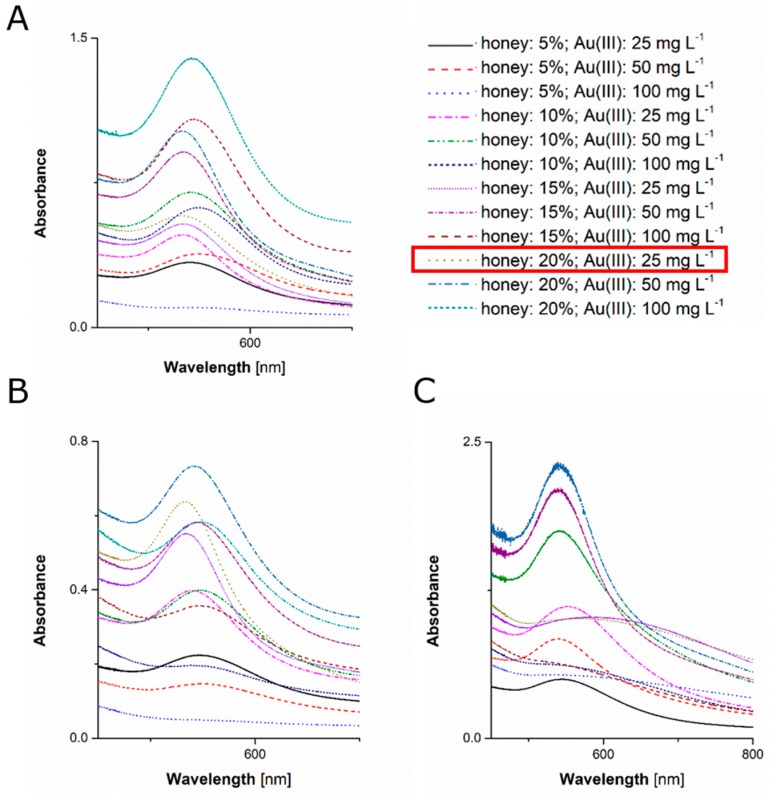
UV/Vis absorption spectra of solutions resulted from mixing solutions of (**A**) lime tree honey, (**B**) multiflower honey, and (**C**) honeydew honey with solutions of Au(III) for green synthesis of AuNPs. The red box indicates pattern of samples subjected to further analytical procedures.

As shown in Figure 2, the LSPR absorption band was identified in UV/Vis absorption spectra of all of mixed solutions. Hence, on the basis of values on the LSPR absorption band λ_max_, it was possible to select optimal conditions for the production of the smallest in size AuNPs (Figure 2). Accordingly, the lowest value of the LSPR absorption band λ_max_ was 538.5 nm and was observed for conditions when the lime tree honey solution was used and its final concentration in the mixed solution was 20% (*w*/*v*) while the concentration of Au(III) ions was 25 mg·L^−1^. When using multi-flower solutions, similar conditions in reference to concentrations of honey and Au(III) ions were also found to produce the smallest in size AgNPs with the LSPR absorption band λ_max_ located at 538.2 nm. By contrast, optimal conditions for the synthesis of AuNPs with honeydew honey solutions related to the concentration of honey and Au(III) in the mixed solution was 5% (*w*/*v*) and 50 mg·L^−1^, respectively. For all honey solutions used, it was established that when the concentration of Au(III) ions in the mixed solution increased, the position of the LSPR absorption band λ_max_ also increased, indicating that the AuNPs higher in size were formed in these conditions (see results in Table 1). Therefore, for all subsequent experiments, AgNPs were synthesized mixing solutions of honeys and Au(III) ions so as their final concentrations in mixed solutions were 20% (*w*/*v*) and 25 mg·L^−1^, respectively. Although these latter conditions were not optimal for honeydew honey, they were applied for consistency with conditions established for two other honeys (lime tree, multi-flower).

### 3.2. Morphology of AuNPs Synthesized under Selected Conditions 

**Figure 3:**
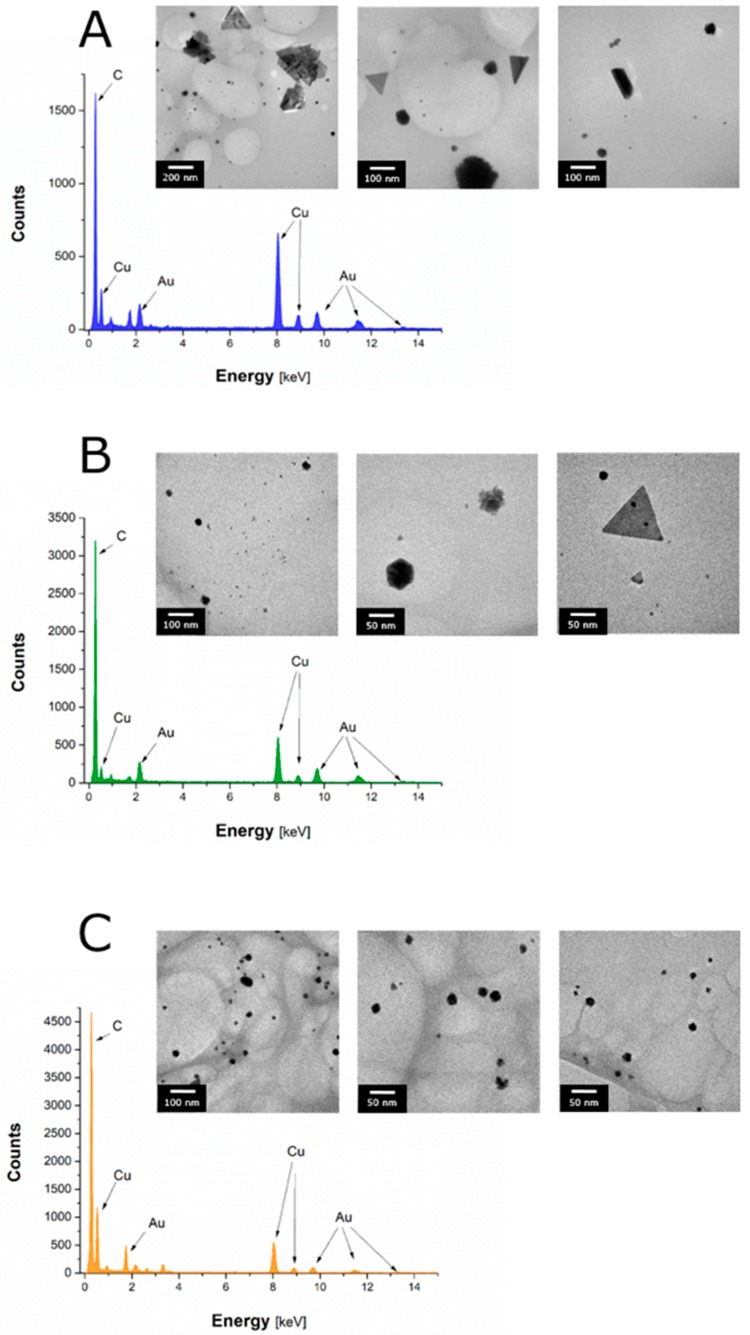
Transmission electron microscopy (TEM) photomicrographs and energy-dispersive X-ray spectroscopy (EDX) spectra of AuNPs produced with the aid of (**A**) lime tree honey, (**B**) multiflower honey, and (**C**) honeydew honey solutions.

TEM was used to assess granulometric properties of AuNPs produced under selected experimental conditions (see Section 3.1 and Figure 2). It was observed that AuNPs synthesized using the lime tree honey solution consisted of aggregated grains (see Figure 3A). A variety of shapes were found, including spherical (68%), triangular (18%), rod-like (5%), trapezoidal (3%), star-like (1%), and polyhedral (5%). Average size of all these AuNPs was 51.6 ± 84.4 nm. AuNPs produced using the multiflower honey solution were well dispersed, uniform in size, and non-aggregated (Figure 3B). Again, a variety of shapes, including spherical (77%), triangular (17%), polyhedral (2%), trapezoidal (2%), hexagonal (1%), and rod-like (1%), were observed for these AuNPs. Their average size was 20.6 ± 23.3 nm and was lower as compared to the average size of Au nanostructures produced with the aid of the lime tree honey solution. Finally, Au nanostructures synthesized using the honeydew honey solution were well dispersed and non-aggregated as well (Figure 3C). Their average size was 25.1 ± 26.3 nm, which was lower as compared to Au nanostructures produced with the aid of the lime tree honey solution, and similar to this obtained with the multiflower honey solution. For honeydew honey, most of AuNPs were spherical (80%), but other shapes, i.e., triangular (2%), polyhedral (17%), and rod-like (1%), were found as well.

EDX was applied to confirm the elemental composition of synthesized AuNPs (Figure 3). As expected, peaks corresponding to Au, C, and Cu (from the Cu grid onto which NFs were placed) were identified in all NFs (Figure 3A–C), confirming AuNPs presence through green synthesis with honey solutions.

**Figure 4:**
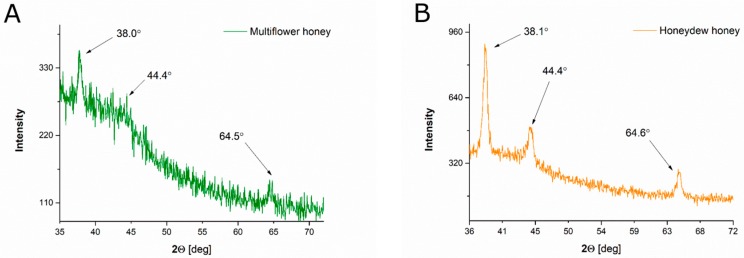
XRD patterns of AuNPs produced by using (**A**) multiflower honey and (**B**) honeydew honey solutions.

XRD was used to determine the crystalline structure of AuNPs produced with the aid of solutions of multiflower and honeydew honeys (Figure 4). In the case of the lime tree honey solution, such analysis was not possible, as the efficiency of AuNPs synthesis appeared to be too low to collect a sufficient amount of dry material. As can be seen in Figure 4, diffraction peaks in diffractograms of analyzed NFs containing AuNPs are situated at 38.1, 44.4, and 64.5° (2θ). Those peaks corresponded to Miller’s indices (111), (200), and (220), respectively, which are intrinsic for the face-centered cubic (fcc) crystalline structure for Au.

To compare the average size of crystalline AuNPs evaluated using TEM and XRD, the Scherrer equation was used. For AuNPs produced with the multiflower honey solution or with the honeydew honey solution, this equation was applied to the width of the most intense diffraction peak in the XRD pattern, i.e., the one with (111) Miller’s indices. Accordingly, it was established that the average size of Au nanostructures produced using the multiflower honey solution was 18.0 nm. In the case of the honeydew honey solution it was 12.2 nm. The average size of AuNPs estimated by TEM was about twice higher than the average size estimated on the basis of XRD measurements. This obvious divergence is reported in literature and attributed to the fact that XRD provides the distribution of the average size of NPs in terms of their volume, while TEM gives distribution of average size by their number [60]. When the number size distribution is calculated, each particle has equal weighting, while the volume size distribution recognizes the average size of the NPs revealing the greatest total volume.

### 3.3. Qualitative Analyses of Chemical Compounds Present in Mixed Solutions

**Figure 5:**
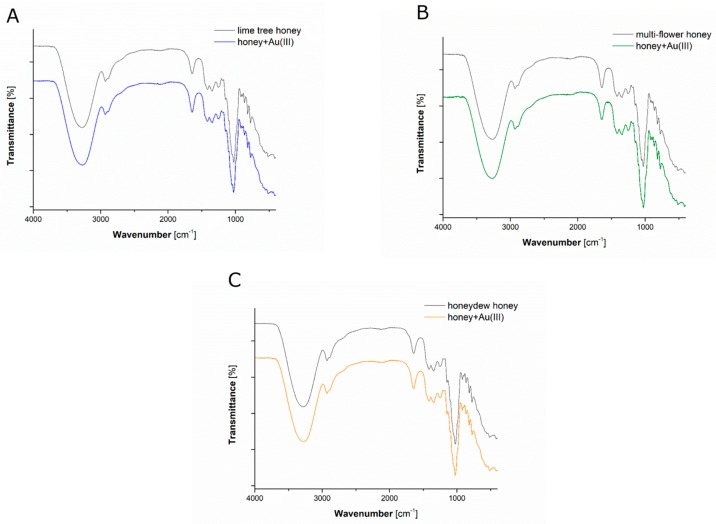
Attenuated total reflectance Fourier transform-infrared spectroscopy (ATR FT-IR) spectra acquired for honey solutions as well as for mixed solutions containing AuNPs that were produced with the aid of (**A**) lime tree honey, (**B**) multiflower honey, and (**C**) honeydew honey solutions.

ATR FT-IR spectroscopy was used to identify chemical compounds present in honey solutions as well as in mixed solutions (Figure 5). Considering ATR FT-IR spectra of honey solutions, the most intense bands (around 3270 cm^−1^) were related to stretching vibrations ν of the –OH group, originating from water molecules (Figure 5). Occurrence of bands at 2930 cm^−1^ was associated with stretching vibrations ν of the C–H group, or with stretching vibrations ν of the N–H group [61]. Mentioned stretching vibrations ν might have originated from carboxylic acids or from free amino acids, respectively, present in all analyzed honey solutions [61]. Furthermore, bending vibrations δ of the O–CH group (range 1342–1418 cm^−1^) were related to carbohydrate compounds like glucose and D-fructose, also occurring in analyzed honey solutions [61]. In addition, the presence of proteins was confirmed by intense peaks (range 1450–1240 cm^−1^), related to stretching vibrations ν of the C-N group (the amide III band), and in-plane bending vibrations δ of the N–H group (the amide II band). Moreover, proteins were identified due to the occurrence of C–O–C symmetric stretching vibrations ν and C–O–H bending vibrations δ (around 1026 cm^−1^) of the –C=O group [42].

ATR FT-IR spectra of mixed solutions containing AuNPs, produced using given honey solutions, were very similar to those recorded for aqueous honey solutions (Figure 5). However, the identified bands were slightly shifted towards longer wavelengths in case of mixed solutions containing synthesised AuNPs. Those shifts in wavelengths of vibration bands might suggest that carboxylic acids, free amino-acids, monosaccharaides (glucose, D-fructose), and proteins present in honey solutions could be responsible for the stabilization of resultant AuNPs [42].

### 3.4. Quantitative Analyses of Reducing Sugars and Phenolic Compounds Contained in Honeys

**Figure 6:**
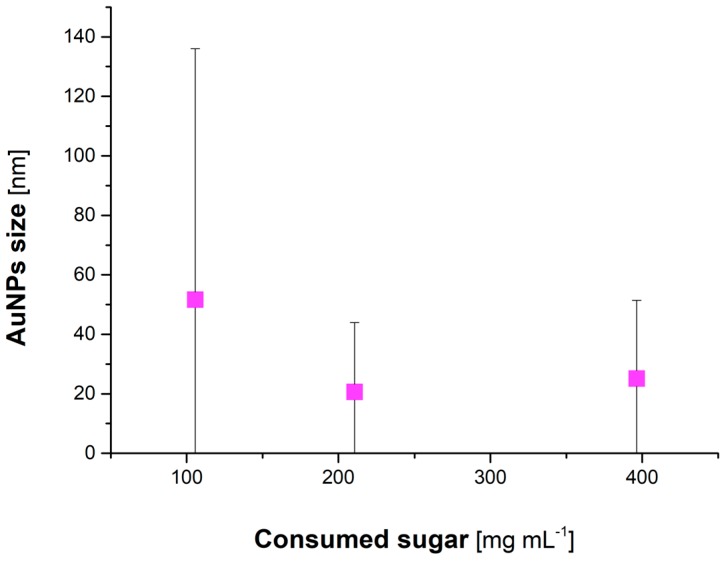
Relationship between the AuNPs size and the concentration of consumed reducing sugars (mg·mL^−1^).

Several studies demonstrate that reducing sugars and phenolic compounds play a significant role in green synthesis of AuNPs [28,33,55]. Their contribution to the formation of AuNPs is likely associated with their reducing and stabilizing properties [55]. For that reason, the concentration of reducing sugars and phenolic compounds in honey solutions used for green synthesis of AuNPs, before and after the addition of a solution of Au(III) ions into them, have been examined. It was found that the concentration of reducing sugars and phenolic compounds in honey solutions changed after the addition of Au(III) ions (Table 2). This suggested that these compounds were involved in the production of Au nanostructures. In addition, a relationship between the concentration of reducing sugars in honey solutions and the average size of produced AuNPs was found (Table 2, Figure 6). Honey solutions with high total concentrations of reducing sugars seemingly led to formation of smaller in size AuNPs. As was suggested by Snitka et al. [43], honey-mediated formation of AuNPs was related to the total concentration of reducing sugars, as well as to the type of reducing sugars. Apparently from the cited work, glucose (aldose) was found to be the main reducing agent in green synthesis of AuNPs, and this was associated with the presence of the aldehyde group [43]. On the other hand, D-fructose (ketose) exhibited only stabilizing properties during synthesis of AuNPs [43]. Stabilizing properties of D-fructose were related to repulsive forces (electrostatic or steric) due to its hydrophobic carbon chain. Overall, Snitka et al. suggested that glucose was a primary compound involved in honey-mediated reduction of Au(III) to Au^0^ of nanometric size, whereas D-fructose behaved as a capping agent for AuNPs nucleation and their growth, and prevented their aggregation [43]. However, no correlation between the total content of phenolics and size of resultant AuNPs was found (Table 2). Thus, it was concluded that reducing sugars were crucial for determining the size of resultant AuNPs.

### 3.5. Application of AuNPs in Microwave-Induced Hyperthermia

Microwave-thermal behaviour of AuNPs obtained with the aid of multiflower and honeydew honeys was tested. Those nanostructures were characterized by the smallest average size, i.e., ~20–25 nm (see Figure 3C). Hence, it was expected that those AuNPs would be the most susceptible to excitation by a microwave radiation field. The obtained data for AuNPs produced with solutions of both mentioned honeys were very similar; therefore, only slight differences between each other were expected.

**Figure 7:**
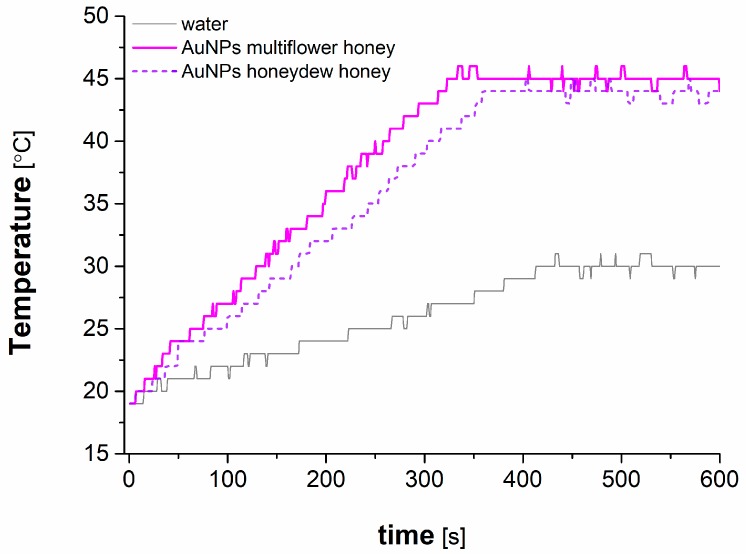
Microwave-induced heating curves recorded for water and the nanofluid containing AuNPs obtained with the aid of a multiflower honey solution.

In Figure 7, heating curves of water and these recorded for the NF containing AuNPs obtained with the aid of both honeys are shown. As can be seen, AuNPs synthesized using multiflower honey were heated to 45 °C in about 5 min. Meanwhile, the AuNPs fabricated in the solution of honeydew honey reached maximum of 44 °C in about 6 min. The differences are attributed to the slightly greater average size of the latter ones, leading to the conclusion, that greater area-to-surface ratio ensures more effective heating. In the same time, exposure to microwaves (at 15 W) led water to be heated up to 30 °C, however, the system needed almost 7 min to achieve this. As a result, the greatest heating rate to maximum temperature was recorded for the medium with AuNPs obtained with the aid of multiflower honey (11.7 × 10^−3^ s^−1^) as compared to this for water (6.8 × 10^−3^ s^−1^). Since the tested NFs with AuNPs was heated to temperatures 14–15 °C greater than the temperature of water, it could be stated that AuNPs indeed might be applicable in hyperthermia treatment procedures. To maintain constant temperature of the NF containing AuNPs required the microwave field to be periodically turned off and turned on. No such operation was required for water, which was constantly exposed to microwave radiation. It must be remembered that in real life conditions, both AuNPs and water in tissues would be heated at the same time by exactly the same exposure to microwaves, whether it would be turned off or turned on. Hence, maintaining constant hyperthermia of AuNPs would inevitably lead to cyclic turning off microwave radiation. This in turn would result in a decrease of temperature of surrounding tissues. Therefore, based on the observed phenomena, and due to the fact that Au nanostructures produced with the aid of honey solutions were likely functionalized by monosaccharaides such as glucose and D-fructose, it could be stated that such green synthesized AuNPs would be suitable for microwave hyperthermia treatment of cancer.

## 4. Conclusions

Three types of commercially available Polish honeys were applied for size-defined green synthesis of NFs containing AuNPs. By selecting the type of honey, and the concentration of honey and Au(III) ions in mixed solutions, appropriate experimental conditions mediating green synthesis of the smallest in size Au nanostructures were found. Based on UV/Vis and TEM measurements, the smallest in size AuNPs were efficiently produced when the concentration of honeydew honey and Au(III) ions in the mixed solution was 20% (*w*/*v*) and 25 mg·L^−1^, respectively. Resultant Au nanostructures were mostly spherical in shape (~80%) and exhibited the average size of 20.6 ± 23.3 nm, as assessed by TEM. Elemental composition of so-prepared AuNPs was determined by EDX, while their crystalline structure was established using XRD. It was confirmed that AuNPs were formed with the face-centered cubic crystalline structure. In addition, it was established that NFs containing AuNPs produced by this simple and fast green synthesis method developed here might be useful in microwave-induced hyperthermia treatment of cancer. To reveal the role of chemical compounds present in honey solutions used for synthesis, qualitative and quantitative analyses of chemical compounds likely involved in AuNPs production were carried out. Based on ATR FT-IR measurements, it was suggested that carboxylic acids, free amino-acids, monosaccharaides (glucose, D-fructose), and proteins might be involved in capping and functionalization of produced nanostructures. In addition, it was noted that reducing sugars as well as polyphenolic compounds played a crucial role in AuNPs formation.

## Figures and Tables

**Table 1:** Wavelengths of localized surface plasmon resonance (LSPR) absorption band at its maximum (λ_max_) and their absorbances (A) for AuNPs produced under different experimental conditions.

No.	Type of Honey	Honey Concentration (%)	Au(III) Concentration (mg·L^−1^)	λ_max_ (nm)	A
**1**	Lime tree	20	100	563.4	0.17
**2**	Lime tree	20	50	550.4	0.55
**3**	Lime tree	20	25	538.5	0.43
**4**	Lime tree	15	100	552.5	0.45
**5**	Lime tree	15	50	543.2	0.38
**6**	Lime tree	15	25	540.2	0.21
**7**	Lime tree	10	100	558.1	0.27
**8**	Lime tree	10	50	551.4	0.26
**9**	Lime tree	10	25	539.5	0.20
**10**	Lime tree	5	100	565.2	0.02
**11**	Lime tree	5	50	561.9	0.13
**12**	Lime tree	5	25	550.5	0.12
**13**	Multiflower	20	100	558.3	0.15
**14**	Multiflower	20	50	550.3	0.23
**15**	Multiflower	20	25	538.3	0.25
**16**	Multiflower	15	100	559.1	0.08
**17**	Multiflower	15	50	553.1	0.20
**18**	Multiflower	15	25	538.5	0.21
**19**	Multiflower	10	100	538.4	0.25
**20**	Multiflower	10	50	559.1	0.08
**21**	Multiflower	10	25	553.4	0.19
**22**	Multiflower	5	100	561.4	0.02
**23**	Multiflower	5	50	557.2	0.13
**24**	Multiflower	5	25	547.9	0.14
**25**	Honeydew	20	100	569.6	0.01
**26**	Honeydew	20	50	558.2	0.07
**27**	Honeydew	20	25	550.2	0.90
**28**	Honeydew	15	100	654.1	0.16
**29**	Honeydew	15	50	576.9	0.04
**30**	Honeydew	15	25	657.2	0.25
**31**	Honeydew	10	100	595.1	0.06
**32**	Honeydew	10	50	553.5	0.59
**33**	Honeydew	10	25	551.3	0.39
**34**	Honeydew	5	100	631.8	0.04
**35**	Honeydew	5	50	540.7	0.83
**36**	Honeydew	5	25	552.3	0.28

**Table 2:** Total concentration of reducing sugars (in mg·mL^−1^) and phenolic compounds (expressed as a gallic acid equivalent in mg·L^−1^) determined in undiluted honeys before and after addition of Au(III) ions for AuNPs synthesis.

Honey	Before Addition of Au(III)		After Addition of Au(III)		AuNPs Mean Size (nm)
	Reducing Sugars	Polyphenols	Reducing Sugars	Polyphenols	
Lime tree	620.7	65.97	515.0	56.29	51.6 ± 84.4
Multiflower	780.2	41.61	569.6	35.46	20.6 ± 23.3
Honeydew	889.5	145.4	493.1	104.7	25.1 ± 26.3
